# Natural History of Uterine Fibroids: A Radiological Perspective

**DOI:** 10.1007/s13669-018-0243-5

**Published:** 2018-05-08

**Authors:** Srirupa Ghosh, Joel Naftalin, Rachel Imrie, Wee-Liak Hoo

**Affiliations:** 1 King’s Fertility, King’s College Hospital, Unit 6, Business Park, Denmark Hill, London, SE5 9RS UK; 2 University College Hospital, 235 Euston Road, Fitzrovia, London, NW1 2BU UK

**Keywords:** Fibroids, Leiomyoma, Fibroid growth, Pregnancy, Inter-gravid, Life cycle

## Abstract

**Purpose of Review:**

This review aims to update our understanding of the radiological life cycle of fibroids, so that we may better counsel patients making difficult treatment decisions. Evidence for both pregnant and non-pregnant women have been considered separately.

**Recent Findings:**

Recent findings have shown that fibroids can undergo both growth and regression in non-gravid uterus. In pregnant women, fibroid growth is non-linear fashion, with the greatest growth occurring in the first 7 weeks of pregnancy. Growth in the later trimesters was significantly slower.

**Summary:**

Fibroid growth, both in the intra- and inter-gravid states, is variable and can range from 18 to 120% per year. In the inter-gravid state, fibroids can grow or undergo spontaneous regression. Factors that can predict fibroid growth include the starting volume of fibroid, type of fibroid and age of patient. In the gravid state, fibroids appears to grow in a non-linear pattern, with the most rapid growth occurring in the first trimester. Factors affecting fibroid growth in pregnancy include the size of fibroids and number of fibroids.

## Introduction

Uterine fibroids (leiomyomas) are the most common gynaecological tumours affecting premenopausal women [1]. They are benign, hormonally dependent tumours arising from the myometrial layer of the uterus [2•]. Prevalence of fibroids ranges between 3.3 and 77% [3, 4] and varies with age and ethnicity [5].

Although most women with fibroids are asymptomatic, fibroids are a major source of gynaecological morbidity and have a substantial impact on women’s health and quality of life [6]. The most common symptoms are menorrhagia (heavy menstrual bleeding) and dysmenorrhoea (menstrual pain). The current management of fibroids includes medical therapy (e.g. ulipristal acetate), minimally invasive procedures (e.g. uterine artery embolisation) and surgery (e.g. myomectomy, hysterectomy). Despite the range of treatment options, surgery remains the mainstay of therapy.

Fibroid tissue is comprised mainly of extracellular matrix and characterised by a low mitotic index and is usually considered slow growing [7]. However, some fibroids can grow very large—up to 30 cm^3^ [1].

It is well known that fibroids are sensitive to oestrogens, which may act to grow or maintain their size [8]. Oestrogen, progesterone and epidermal growth factor (EGF) are considered essential to the formation of fibroids [1]. Due to their hormonal nature, it was originally thought that uterine fibroids would develop after puberty, continue to grow in a consistent manner during reproductive life and shrink after the menopause. Despite recent advances in imaging, our understanding of the natural life cycle of fibroids remains poor, which makes counselling difficult.

## Radiological Life Cycle of Fibroids in the Inter-Gravid State

### Variability in Growth of Fibroids in the Inter-Gravid State

It was originally thought that fibroids had a linear growth during a woman’s reproductive life. However, there is evolving evidence that this is not always the case and typically the life cycle of a fibroid may involve growth as well as regression. Indeed, Peddada et al. tracked the growth of 262 fibroids (range 1.0 to 13.0cms) in 72 premenopausal women by performing four MRI scans over a period of 12 months [9]. They found a median growth rate for fibroids was 9.0% per 6 months (range − 89.0% to + 138.0%). Spontaneous regression, defined by a reduction in fibroid volume of greater than 20%, occurred in 7.0% of fibroids. Rapid growth, defined by an increase in volume of greater than 20%, occurred in 34.0% of fibroids. The authors also found that different fibroids within individuals grow at different rates, despite a uniform hormonal milieu [9].

These findings were in agreement with another study by Mavrelos et al., who used ultrasound as their imaging modality [10••]. One hundred twenty-two women underwent a minimum of two transvaginal ultrasound assessments, all performed by a single operator over a nine-year period. The median scan interval between the initial and final ultrasound was 21.5 months (range 8–90 months). Fibroids were measured in three perpendicular planes and the position of every fibroid was recorded. The authors concluded that fibroids underwent both growth and regression despite the maintenance of a stable hormonal environment in premenopausal women. The volume of the largest fibroid increased by a median of 35.0% a year. Whilst, 21.3% (26/122) fibroids appeared to have regressed spontaneously [10••].

In a separate study by Baird et al., MRI assessments of 101 fibroids were made in 18 black and 18 white women at enrolment, 3, 6 and 12 months interval [11]. The median growth rate of fibroids was found to be 7.0% per 3 months. Growth spurts, defined as a greater than or equal to 30% increase over 3 months, were found in 36.6% (37/101) of fibroids. Conversely, shrinkage spurts were seen in 1.0% (1/101). 62.4% (63/101) of fibroids did not demonstrate a growth or shrinkage spurt [11].

Tsuda et al. proposed the use of fibroid vascularity with transvaginal and transabdominal ultrasound as a predictor of fibroid growth [8]. The authors measured fibroid volume and attempted to identify a leiomyoma artery for each fibroid. The participants were reassessed 3 monthly for a year. Fibroid volume increased in 46.2% (24/52) of patients with a leiomyoma artery, compared with 6.1% (3/49) when a leiomyoma artery was not identified (*n* = 52, *P* < 0.001). The authors concluded that the presence of leiomyoma artery can be a useful predictor of fibroid growth [8].

## Factors Affecting Fibroid Growth

### Starting Volume of Fibroid

Mavrelos et al. found that the size of fibroids at presentation significantly influenced their subsequent growth rates [10••]. Small fibroids, defined by a mean diameter of less than 20 mm, increased in size by a median of 51.3% (IQR, 9.3–210.3) per year, whilst large fibroids (diameter of more than 50 mm) increased by a median of 40.7% (IQR, 14.1–67.0) per year. Intermediate fibroids (between 20 and 50 mm) only increased in size by a median 16.8% (IQR, − 7.4–63.0) per year (*P* = 0.007). Fibroids classified as either small or large demonstrated a growth rate nearly three times more than fibroids classed as intermediate in size at presentation [10••].

Similarly, Baird et al. found that the size of the fibroid at initial assessment was a significant variable in predicting growth rates [11]. However, the authors found that large fibroids (≥ 50 mm) were significantly less likely to have short-term variability in growth when compared to small fibroids (< 50 mm) (*P* < 0.001) [11].

Peddada et al. did not find a significant difference in the growth rates of fibroids when fibroids < 14.0 cm^3^ were compared with larger fibroids (*P* = 0.48) [12]. Similarly, Tsuda et al. had the same conclusion when comparing fibroids larger or smaller than 31.9 cm^3^ [8].

### Type of Fibroid

Tsuda et al. found that submucous fibroids were less likely to increase in size. 15.4% (2/13) of submucous fibroids increased in size per year on ultrasound, compared with 27.4% (17/62) of intramural fibroids and 30.7% (8/26) subserous fibroids (*P* < 0.05). This was independent of whether a leiomyoma artery was identified [8].

Conversely, Mavrelos et al. found that intramural fibroids grew the fastest, followed by subserous then submucous fibroids [10••]. Intramural fibroids increased in volume by a median of 53.2% (IQR, 11.2–217.0) per year, subserous fibroid increased by 25.1% (IQR, 1.1–87.1) per year and submucous fibroids increased by 22.8% (IQR, − 11.7 to 48.3) per year (*P* = 0.012). However, due to a disproportionate number of small intramural fibroids, the authors concluded that fibroid growth rate appeared to be independently influenced by the fibroid size, rather than a true reflection of the effect of growth rate on fibroid position [10••].

### Age

Fibroid growth tends to decline with age. Mavrelos et al. found that women less than or equal to 35 years old had a median fibroid of growth rate of 69.1% (IQR, 8.3–185.1) per year, whilst women more than 35 years of age had a median fibroid growth rate of 29.8% (IQR, 0.0–78.7) (*P* = 0.113) [10••].

A similar decline was also reported by Peddada et al.; however, this did not occur for all races [9]. In their study, only white women ≥ 45 years of age demonstrated a significant decline in growth rate, − 19.57% (95% CI, − 30.48 to 6.94) per 6 months when compared white women < 35 years old (*P* = 0.04). For black women ≥ 45 years of age, there was no significant difference in fibroid growth rate compared to black women who were < 35 years old, − 3.38% (95% CI, 17.31 12.90) (*P* = 0.67). Furthermore, fibroids from white women ≥ 45 years of age grew more slowly than those from older black of the same age (2% vs. 15% growth rate, respectively) [9].

### Number of Fibroids

Peddada et al. found that women with solitary fibroids appeared to have a faster growth rate, 33.75% (95% CI, 7.58–61.17) per 6 months when compared to women with multiple fibroids (*P* = 0.06) [9]. However, only 6.9% (5/72) of women had solitary fibroids in this study. The influence of the number of fibroids on growth rates was not commented on other authors [10••, 11].

### Radiological Life Cycle of Fibroids in the Gravid State

The incidence of fibroids in pregnancy is estimated at 0.1 to 10.7% [13, 14, 15••]. The conventional belief is that fibroids grow in a consistent manner during pregnancy due to a number of factors: the increasing steroid hormone levels, hypertrophy and oedema and stretching of the myometrium. Conversely, expansion of the amniotic cavity and ischemia-driven degenerative changes may lead to shrinkage or disappearance of fibroids.

A recent systematic review concluded that fibroids do appear to have a linear growth pattern in the first trimester of pregnancy. However, the growth patterns in later gestations remain contentious with inconsistent evidence [16]. There are also increasing reports that fibroids often remain unchanged or even decrease in size during pregnancy [17, 18].

### Non-linear Growth of Fibroids in Pregnancy

Ciavenetti et al. performed prospective scans on 109 women at four intervals, before pregnancy (scan 1), 7–8 weeks (scan2), 10–13 weeks (scan 3) and 20–22 weeks (scan 4) [15••]. The authors found that of the fibroids measuring 10 to 50 mm at scan 1, 76.9% (110/143) increased in volume between scan 1 and 2, 72.0% (103/143) increased between scan 2 and 3 and 40.6% (58/143) increased between scan 3 and 4. The median growth rate was 122% between scan 1 and 2, 108% between scan 2 and 3 and 25% between scan 3 and 4. The authors concluded that most fibroids increase in size in the first 7 weeks of pregnancy; however, growth during pregnancy was non-linear as growth in the later trimesters was slower [15••].

Further suggestions of non-linear fibroid growth in pregnancy was found by De Vivo et al. who performed three ultrasound scans at 12 (scan 1), 21 (scan 2) and 33 weeks (scan 3) gestation [13]. The mean (± standard deviation) fibroid volume was 17.04 cm^3^ (± 29.1) at scan 1, 38.8cm^3^ (± 67.8) at scan 2 and 42.1 cm^3^ (± 69.0) at scan 3 (*P* < 0.05). The authors concluded that fibroids increased in size throughout pregnancy but growth rates were significantly greater in the first half of pregnancy as compared with the second half of pregnancy [13].

In support of rapid fibroid growth in early pregnancy, Benaglia et al. compared fibroid growth rates between a pregnant cohort (*n* = 46) and a non-pregnant control cohort (*n* = 41), following in vitro fertilisation (IVF) treatment [19]. Ultrasound assessments for fibroid volume and diameter were performed at the start of IVF stimulation and 5 weeks after embryo transfer in both cohorts. The median change of the diameter of all fibroids in pregnant women was + 34% and in non-pregnant women was + 2% (*P* < 0.001). The median change in volume of all fibroids in pregnant women was + 140% and in non-pregnant women 0% (*P* < 0.001). The authors concluded that fibroids more than double in size within 7 weeks of pregnancy [19].

Strobelt et al. performed regular ultrasound fibroid assessments on 134 pregnant women from 16 weeks gestation until term [20]. The authors found that overall, fibroid growth was only found in 14.9% (20/134) of pregnant women and the remaining 85.1% of women had no change in fibroid size after 16 weeks of pregnancy [20].

## Factors Affecting Fibroid Growth in Pregnancy

### Starting Size of Fibroid

Similar to non-pregnant women, the initial size of fibroid appears to influence growth rates. Ciavenetti et al. found in a logistic regression analysis that the odds ratio (OR) for increase in fibroid volume between scan 1 and 2 was 0.97 (95% CI 0.95–1.0) (*P* = 0 .04), between scan 2 and 3 [OR 0.93 (95% CI 0.89–0.97)] (*P* < .001) and between scan 3 and 4 [OR = 0.95 (95% CI 0.92–0.99)] (*P* < .001) [15]*.* The authors concluded that fibroids with smaller starting volumes were more likely to increase in size than larger ones during pregnancy [15].

Strobelt et al. found that after 16 weeks gestation, 75.0% (69/92) fibroids less than 5 cm in diameter resolved (could not be identified or distinguished from surrounding myometrium) during pregnancy, compared with only 35.7% (15/42) of fibroids greater than 5 cm in diameter *(P* < 0.001) [20]. The authors concluded that this may be a result of crowding of the pelvis in advanced gestations or due to changes in the fibroid echogenicity and stretching of the myometrium in advanced gestations.

Other authors had found that there was no correlation between initial fibroid volume and fibroid growth during pregnancy [13].

Laughlin et al. performed a cohort study, using transvaginal ultrasound assessment in early pregnancy and at 3–6 months post-partum on 171 women, with a single fibroid [21]. 36.0% (62/171) of fibroids had resolved beyond the authors’ ability to detect them at the post-partum ultrasound examination. 109/171 fibroids remained detectable on the post-partum ultrasound. However, 79.0% of these fibroids were significantly smaller than on the original early pregnancy scan (median reduction in diameter of 0.5 cm (*P* < 0.001)). Infarction of uterine fibroids secondary to uterine involution in the post-partum period is thought to facilitate this complete regression of small fibroids in the post-partum period [22]. This is further supported by epidemiologic data that suggest that parity is protective against incidence and further development of fibroids [23].

### Number of Fibroids

Strobelt et al. reported that women with solitary fibroids were more likely to resolve when compared to women with multiple fibroids, 73.7% (70/95) and 35.9% (14/ 39), respectively (*P* < 0.001) [20]. There was no correlation with pain which may indicate fibroid degeneration/necrosis during pregnancy that could perhaps explain the apparent disappearance.

Other studies did not replicate these findings [13, 15••, 19].

## Conclusions

Despite fibroids being the most common uterine tumour, their life cycle remains poorly understood. The original teaching dictates that fibroids grow in a linear pattern, starting in puberty and continues through life until the hormonal milieu of the woman changes dramatically at menopause when shrinkage was expected. A review of current evidence suggests that fibroid growth is variable and can range from 18 to 120% per year. Fibroids also undergo spontaneous regression, growth and shrinkage spurts outside of menopause, despite a stable hormonal environment. There is conflicting evidence regarding factors that affect fibroid growth. Many studies have investigated the impact of size at presentation; however, there is no agreement as to whether smaller or larger fibroids grew faster. With regard to the position of fibroids in relation to the uterine cavity, submucous fibroids were least likely to increase in size. Whilst reduced growth rates have been reported with increasing age, this trend was not statistically significant especially in white women.

Recent evidence would suggest a non-linear growth pattern during pregnancy with during pregnancy with significantly significantly increased growth rates in the first half of pregnancy, especially in the first trimester. The starting volumes of fibroids have been found to affect fibroid growth; however, there was no consistent evidence of their effects. Solitary fibroids in pregnancy were more likely to resolve when compared to multiple fibroids.

It would be ideal if we were able to counsel women regarding the specific timing of various medical and surgical interventions and their outcomes in pregnancy. However, this would require further research into the life cycle of fibroids and further understanding of growth variables, both in the inter-gravid and gravid states. Recent evidence has challenged traditional views and provided valuable insight into the variability of the radiological life cycle of fibroids, but more evidence is needed so that gynaecological and obstetric treatments can be optimised.
